# Advances in bedside imaging: lung ultrasound

**DOI:** 10.1186/s40635-025-00838-5

**Published:** 2025-12-11

**Authors:** Elina Nazarian, Jante S. Sinnige, Lieuwe D. J. Bos, Marry R. Smit

**Affiliations:** 1 Department of Intensive Care, Amsterdam UMC, University of Amsterdam, Meibergdreef 9, 1105 AZ Amsterdam, The Netherlands; 2 Laboratory of Experimental Intensive Care and Anaesthesiology (L.E.I.C.A.), University of Amsterdam, Amsterdam, The Netherlands

**Keywords:** Lung ultrasound, Acute respiratory failure, Intensive care, Innovations

## Abstract

Lung ultrasound has become an indispensable tool in the management of acute respiratory failure, offering real-time, radiation-free bedside imaging. Its portability, repeatability, and high sensitivity for detecting pulmonary abnormalities have made it particularly valuable in critical care settings, especially during the Coronavirus disease 2019 pandemic. This narrative review explores the evolving role of lung ultrasound, examining both its established clinical applications and recent advances in artificial intelligence and imaging analysis. These developments emphasize the growing importance of lung ultrasound not only as a diagnostic tool but also as a platform for innovation, with artificial intelligence-driven approaches to further enhance its clinical utility.

## Introduction

Lung ultrasound (LUS) has emerged as a frontline imaging modality in intensive care units due to its diagnostic accuracy, ease of use, and bedside applicability. Unlike chest X-rays (CXR) and computed tomography (CT) scans, LUS is portable and can be performed repeatedly without radiation exposure. It has shown comparable sensitivity and specificity in detecting conditions including pneumothorax, pleural effusion, and pulmonary edema, when compared to expert diagnoses based on CXR and CT imaging [1]. The development of standardized protocols, such as the BLUE (bedside lung ultrasound in emergency) protocol, has further improved diagnostic consistency and reduced operator variability [1]. During the coronavirus disease 2019 (COVID-19) pandemic, LUS played a key role in evaluating and monitoring lung involvement in patients with acute respiratory failure (ARF), supporting both triage and therapeutic decision [2]. These developments have solidified LUS as a valuable extension of the physical examination in critical care.

This review aims to summarize recent advances in the application of LUS in the diagnosis and management of ARF, focusing on both clinical studies and experimental innovations. We will highlight how LUS has evolved from a supportive diagnostic tool to an essential component of critical care, and explore emerging techniques and future directions for research in this field.

## Lung ultrasound principles

LUS differs from conventional imaging modalities such as CT or CXR in that it relies primarily on artefacts and pleural characteristics rather than direct visualization of lung structures.

In this section, we discuss the most important principles in LUS that are used for diagnosis and disease monitoring.

The majority of the LUS device systems can be moved to the patients’ bedside, which eliminates the need for transportation and is particularly useful for critically ill patients. In clinical practice, straight linear array probes are most often used for pleural examination (5–13 MHz), since these probes provide better resolution for identification of superficial structures, including the pleural line. For deeper imaging, (micro)convex or curvilinear array probes are used (1–8 MHz) [3]. The pleura, lungs, and chest wall can all be imaged in brightness-mode (B-mode) imaging. In addition to B-mode, motion-mode (M-mode) can aid the sonographer in detection of lung sliding, a sonographic sign used to exclude pneumothorax [4]. Ultrasound gain settings strongly influence the visibility of pleural characteristics [5]. For consistent imaging, the gain should be adjusted so that the pleural line is clearly visible without excessive artifact noise below it. The pleural line should be positioned at approximately one-third of the screen depth, with the focal point set at the pleural line.

**Fig. 1:**
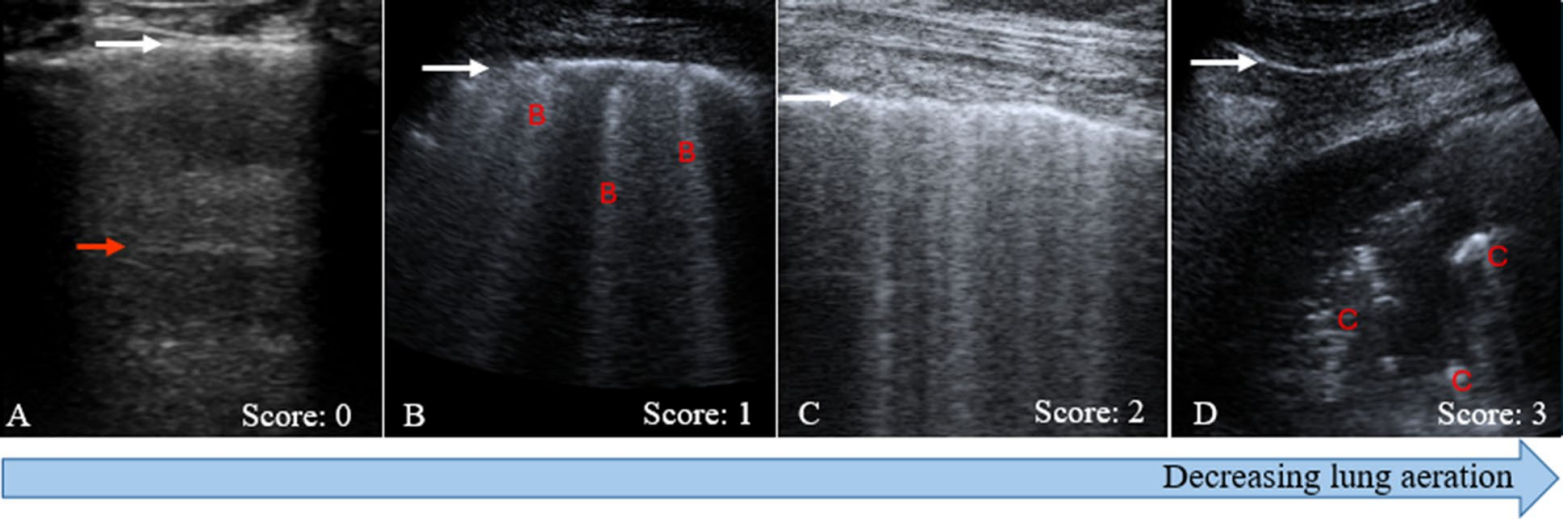
Principles in lung ultrasound (LUS) and profiles. **A** Normally aerated lung (LUS aeration score 0) showing the pleural line (white arrow) and A-line (red arrow) which represents a reverberation artefact of the pleural line. **B** Mild aeration loss (LUS aeration score 1) with three B-lines denoted in red. **C** Moderate aeration loss (LUS aeration score 2) where the B-lines take up more than half of the pleural line. **D** Severe aeration loss (LUS aeration score 3) with consolidations indicated in red

During a LUS exam the pleural line must first be identified. The pleural line appears between and beneath the ribs on a longitudinal scan when placing the probe perpendicular to adjacent ribs. This can be observed in the form of a bat sign, where the hyperechoic pleural line between two adjacent ribs represents the bat’s body and the ribs represent the bat’s wings; see Fig. 1A. This important landmark is used to effectively detect the surface of the lungs. The pleural line moves with respiration during real-time LUS. This finding, known as lung sliding, excludes pneumothorax [6]. The number of zones to scan during LUS differs per protocol [7]. A commonly used protocol consists of six regions per hemithorax; including anterior, lateral, and posterior fields which are divided into superior and inferior regions [7]. To quantify lung aeration, the LUS aeration score is determined, based on the characteristics observed in the LUS examination in the 12 regions. The lung ultrasound aeration score was found to be strongly correlated with lung tissue density on CT [8, 9].

**Fig. 2:**
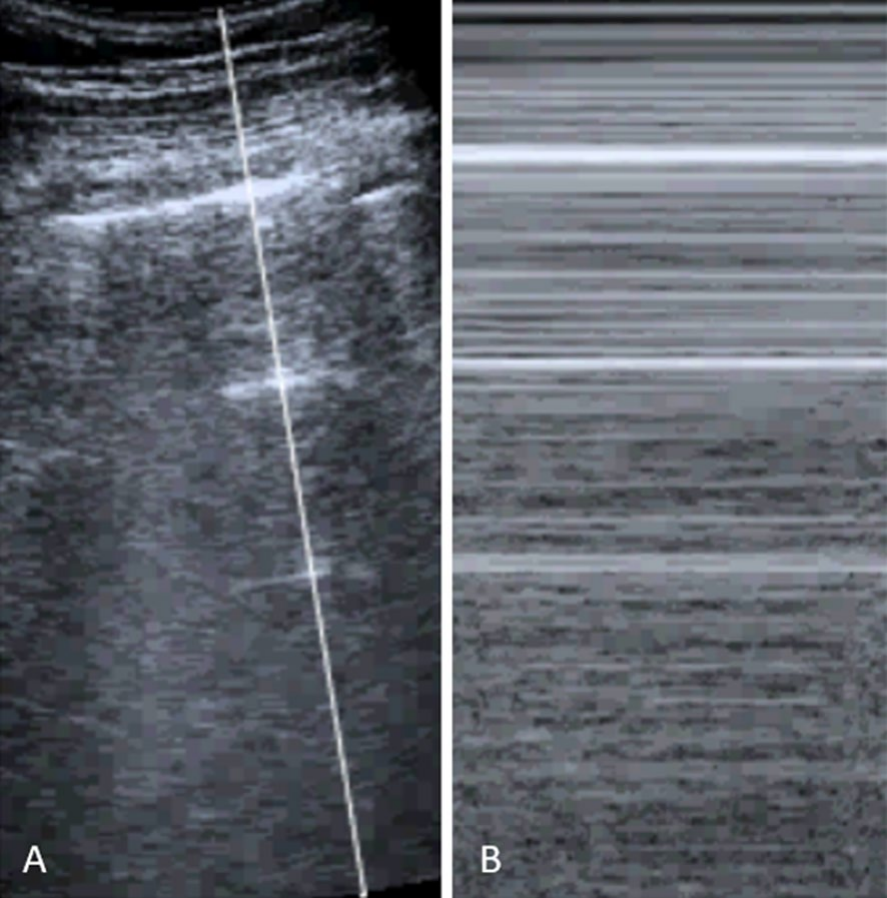
LUS features of pneumothorax. **A** B-mode image showing a pneumothorax with the pleural line and multiple A-lines. **B** M-mode demonstrating absent lung sliding

In normally aerated lung, horizontal A-lines are visible underneath and parallel to the pleural line (Fig. 1A). These are hyperechoic lines between the pleural line and the bottom of the screen that are created because of reverberation artefacts. B-lines are long vertical hyperechoic lines from the pleural line extending to the bottom of the screen, obscuring A-lines (Fig. 1B). These line are associated with thickening of the interlobular septa, which can be caused by edema or fibrosis. For the LUS aeration score, zero points are assigned for regions with horizontal A-lines with two B-lines at the most (normal aeration), 1 point is assigned if more than two regularly spaced B-lines are observed that take up less than half of the scan (mild loss of aeration). A LUS aeration score of 2 points is assigned when multiple B-lines cover more than half of the scanned pleural line (moderate loss of aeration, Fig. 1C) [7]. Lung consolidations, which indicate severely reduced aeration, appear on LUS as hypoechoic, heterogeneous echostructures (Fig. 1D) and are assigned a LUS aeration score of 3. The presence of non-aerated lung tissue is a crucial sign for diagnosis of pneumonia and atelectasis [6, 7, 10]. Pleural effusion usually appears as a homogeneous anechoic fluid-filled structure. When the effusion is large, it can give rise to compressive atelectasis of the adjacent lung, which can be observed as a tissue-like structure [11]. Figure 2 illustrates features of a pneumothorax (Fig. 2A), including the absence of lung sliding observed in M-mode (Fig. 2B).

## Clinical applications

### Diagnosis

**Fig. 3:**
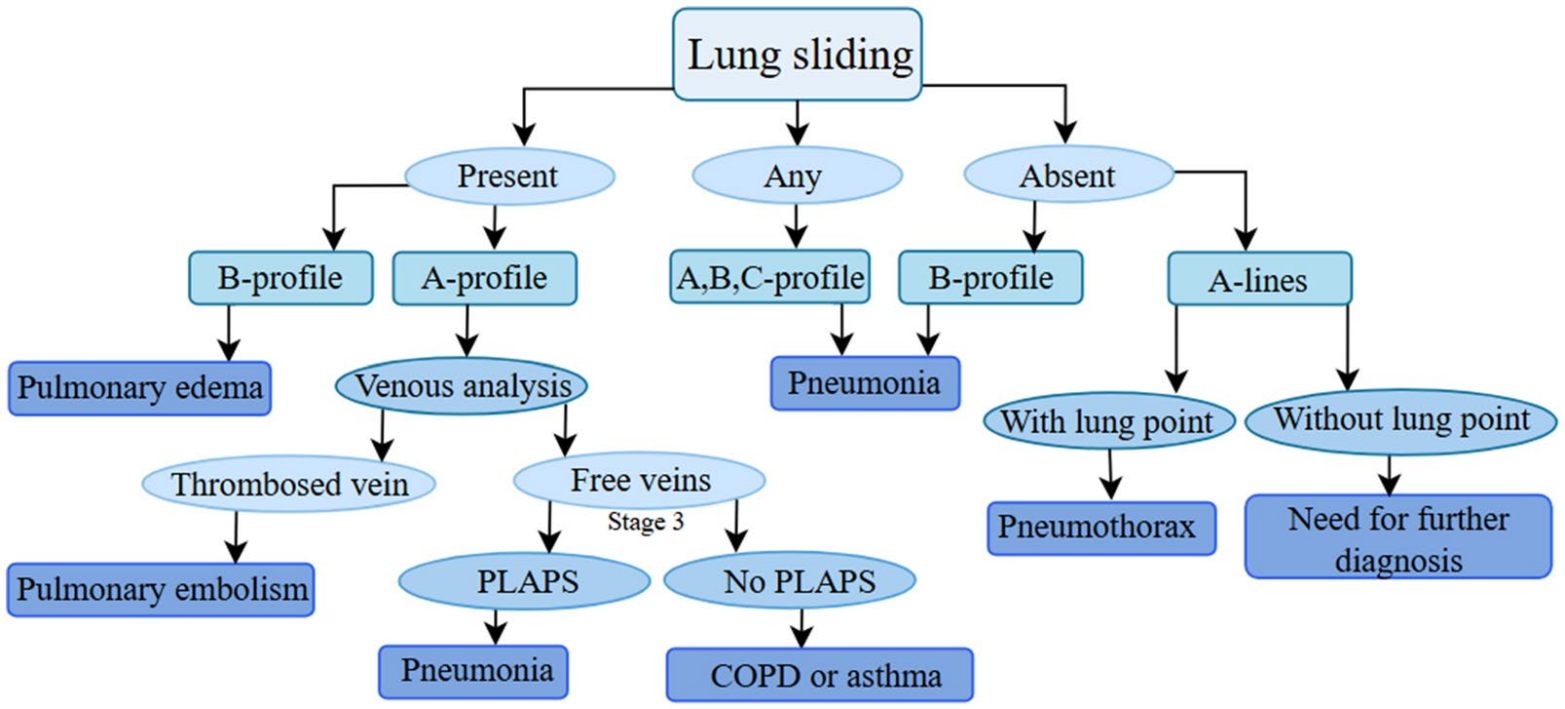
Diagnostic scheme according to the BLUE protocol for LUS. Scheme illustrating the BLUE protocol developed by Lichtenstein and Meziere [1] for diagnosis using LUS with a 90.5% accuracy. Abbreviations: bedside lung ultrasound in emergency (BLUE), lung ultrasound (LUS), posterolateral alveolar and/or pleural syndrome (PLAPS), chronic obstructive pulmonary disease (COPD)

To identify the cause of ARF, the bedside lung ultrasound in emergency (BLUE) protocol [1, 6, 12] assesses three specific examination points on both the left hemithorax and on the right hemithorax; see the diagram in Fig. 3. While not the only protocol available, BLUE is widely used in emergency and critical care settings [1]. In addition to the two BLUE points per hemithorax, the posterolateral alveolar and/or pleural syndrome (PLAPS) point is investigated for alveolar and/or pleural pathology [12]. The severity of lung aeration loss and cause of ARF is determined by the LUS patterns that are observed.

Alveolar-interstitial disease is associated with the presence of lung rockets and a diffuse and patchy pattern [13]. Pneumonia is characterized by the presence of B-lines and lung consolidations with a tissue-like echotexture, resembling the liver. These features are also seen in atelectasis. However, a key distinguishing feature of pneumonia is the presence of air bronchograms that move with respiration (dynamic), whereas in atelectasis, they remain static [14, 15]. In cases of chronic obstructive pulmonary disease (COPD) exacerbation or asthma, respiratory insufficiency is typically associated with a sonographic A-profile pattern with the presence of lung sliding and the absence of PLAPS, posterior-lateral consolidations, or alveolar consolidations. In patients with acute dyspnea or other cases of respiratory failure a B-profile was determined predominantly [16]. Pulmonary edema on lung ultrasound is identified by the presence of multiple B-lines, with three or more B-lines in the anterior or lateral lung regions considered abnormal [7].

#### ARDS

Acute respiratory distress syndrome (ARDS) is characterized by bilateral opacities that are not explained by effusions, lobar/lung collapse, or nodules on imaging. It involves respiratory failure that cannot be attributed solely to cardiac failure or fluid overload, and impaired oxygenation graded by partial arterial oxygen pressure to fraction of inspired oxygen (PaO₂/FiO₂) (≤ 300 mmHg) with positive end-expiratory pressure (PEEP) ≥ 5 cmH₂O [1, 17, 18]. On LUS, ARDS is characterized by a diffuse bilateral B-pattern, often accompanied by subpleural consolidations and an irregular pleural line [1, 17]. This irregular or thickened pleural line can help to distinguish ARDS from hydrostatic or cardiogenic pulmonary edema, which usually presents with a smooth pleural line [17]. The Kigali modification, which was designed for low-resource settings with limited access to imaging and mechanical ventilation, allows bilateral B-lines or consolidations on LUS to fulfill the imaging criteria for ARDS [19]. These criteria showed strong sensitivity but limited specificity when evaluated against the Berlin criteria in high resource setting [20]. The New Global definition incorporated the LUS-based criteria introduced in the Kigali modification, enabling consistent diagnosis in both high- and low-resource environments [21]. A more comprehensive and data-driven approach to LUS diagnosis of ARDS is the LUS-ARDS score, calculated as 2.5 × left LUS aeration score + 1 × right LUS aeration score + 3.5 × number of anterolateral regions with an abnormal pleural line [20]. This score has high diagnostic accuracy compared to consensus ARDS diagnosis by an expert panel.

#### Phenotyping ARDS

Two ARDS sub-phenotypes have been described via CT imaging: focal, with dorsal-inferior consolidations, and non-focal, with diffuse and patchy aeration loss [22]. These phenotypes respond differently to ventilation strategies. Focal ARDS patients tend to benefit from lower PEEP and prone positioning, whereas recruitment maneuvers and higher PEEP have been demonstrated to improve lung mechanics in non-focal ARDS patients. Incorrect phenotyping can lead to suboptimal ventilation and worse outcomes. While CT remains the reference standard for phenotyping, its feasibility is limited due to transport risks, cost, radiation exposure, and need for substantial expertise [22]. LUS offers a promising alternative, with classification methods based on aeration scores showing high specificity, sensitivity, and accuracy. Importantly, the anterior lung zones contribute most to morphological classification [23, 24]. An ongoing prospective study, the PEGASUS trial, is assessing the benefit of implementing personalized ventilation strategies based on LUS-derived morphological sub-phenotype classification [25, 26]. In this trial, a patient is classified as having non-focal lung morphology if the anterior LUS score is ≥ 2, or if the lateral LUS score exceeds the posterior LUS score in either lung. Focal morphology is assigned when neither of these conditions is met.

#### LUS compared to other imaging methods

LUS is low-cost, portable and fast. When performed properly, LUS has higher accuracies compared to CXR for detection of pneumonia [27], pneumothorax [28], pulmonary edema [29], pleural effusion, and lung consolidations [30]. See Table 1 for sensitivity, specificity, and area under the receiver operating characteristics curve (AUROCC) values of meta-analyses comparing LUS and CXR, in which CT scans were used as a reference.

**Table 1:** Diagnostic performance for CXR vs LUS

Pathology	Test	Sensitivity	Specificity	AUROCC
Pneumonia [27]	CXR	0.77 (CI: 0.73–0.80)	0.91 (CI: 0.87–0.94)	0.91
	LUS	0.95 (CI: 0.93–0.97)	0.90 (CI: 0.86–0.94)	0.97
Pneumothorax [28]	CXR	0.47 (CI: 0.31–0.63)	1.00 (CI: 0.97–1.00)	
	LUS	0.91 (CI: 0.85–0.94)	0.99 (CI: 0.97–1.00)	
Cardiogenic	CXR	0.77 (CI: 0.67–0.84)	0.87 (CI: 0.79–0.92)	
Pulmonary edema [29]	LUS	0.92 (CI: 0.81–0.97)	0.92 (CI: 0.87–0.96)	
ARDS [31]	CXR	-	-	
	LUS	0.63 (CI: 0.45–0.78)	0.94 (CI: 0.86–0.98)	0.88
Consolidation [30]	CXR	0.53 (CI: 0.35–0.70)	0.78 (CI: 0.53–0.91)	0.69
	LUS	0.92 (CI: 0.78–0.97)	0.92 (CI: 0.70–0.98)	0.96
Pleural effusion [30]	CXR	0.42 (CI: 0.32–0.53)	0.81 CI: 0.67–0.90)	0.57
	LUS	0.91 (CI: 0.83–0.96)	0.92 (CI: 0.82–0.97)	0.96

Overview of meta-analyses reporting diagnostic performance of CXR versus LUS, including sensitivity, specificity with corresponding 95% CI, and AUROCC if available. CT served as the reference standard in most analyses, except for cardiogenic pulmonary edema, where medical records were used as the reference. Abbreviations: chest X-ray (CXR), confidence intervals (CI), area under the receiver operating characteristics curve (AUROCC), computed tomography (CT)

### Disease monitoring and management

#### Lung recruitment

In acute lung injury, recruitment of collapsed lung tissue can be achieved through increased PEEP or the application of recruitment maneuvers, aimed at restoring lung aeration. This leads to improved gas exchange and respiratory mechanics by increasing the number of alveoli participating in tidal ventilation. The gold standard for assessment of lung recruitment is CT imaging, which quantifies non-aerated tissue that becomes re-aerated at higher airway pressures [32, 33]. LUS offers a non-invasive, radiation-free bedside alternative to guide and assess recruitment. Using the LUS re-aeration score, ultrasound can estimate lung recruitment, as measured with the pressure–volume curve method [34]. However, the changes in LUS aeration scores after recruitment were not found to correlate with lung recruitment as measured by CT scan [8, 34]. A challenge in using LUS to guide recruitment strategies has been the difficulty in detecting overdistension in mechanically ventilated patients. Recent research suggests that spatiotemporal pleural line analysis may help identify changes in lung sliding as PEEP increases [35]. This approach could allow for earlier detection of overdistension, reducing the risk of ventilator-induced lung injury.

#### Prone positioning

In addition to recruitment maneuvers, prone positioning is another strategy used to improve oxygenation, ventilation–perfusion ratio [36] and mortality rates [37] in ARDS patients. It has been demonstrated to be specifically beneficial in the focal phenotype [22]. Studies indicate that LUS can help to predict the response to prone positioning and assess prognosis in ARDS patients [38–40]. This was demonstrated by a reduced LUS aeration score after prone positioning, with survivors showing a greater reduction in the LUS aeration score [38]. Notably higher anterior LUS scores (indicating lower anterior aeration) were associated with a poorer improvement in PaO₂/FiO₂ after prone positioning [40].

#### Fluid management

Pulmonary edema is independently linked to higher mortality, prolonged mechanical ventilation, and longer intensive care unit (ICU) stay [41]. Therefore, restrictive fluid management is essential in critically ill patients. While tools like pulmonary artery catheters and pulse contour analysis offer guidance, they are invasive and costly [42]. CXR, though more accessible, lack sufficient accuracy and involve radiation exposure [43]. In contrast, LUS provides a non-invasive and accurate method to detect pulmonary edema. LUS-guided deresuscitation is currently being evaluated in a prospective randomized trial, comparing its effectiveness to routine care in invasively ventilated ICU patients [44].

#### Weaning

One common cause of weaning disruption is fluid accumulation in the lungs during spontaneous breathing trials, known as weaning-induced pulmonary edema (WIPO). This can be detected using LUS, where an increase of six or more B-lines, specifically in anterior and lateral pulmonary fields, during the breathing trial indicates a high likelihood of WIPO [45, 46]. A LUS aeration score above 17 points has been associated with weaning failure due to impaired pulmonary aeration, both during the weaning process and the post-extubation period; whereas, a LUS aeration score below 13 has been associated with successful weaning [47]. Other causes of weaning failure, including cardiovascular dysfunction, diaphragmatic weakness, respiratory insufficiency, and airway obstruction, can also be identified using LUS [48]. Bedside LUS assessments during weaning have been proposed to help reduce the duration of mechanical ventilation and ICU stays, as well as to prevent post-extubation respiratory failure that may necessitate reintubation, ultimately aiming to reduce mortality risk [49].

#### Prognostication

Prognostication plays a crucial role in managing critically ill patients with ARF, enabling clinicians to predict outcomes, guide therapeutic decisions, and optimize resource use. LUS, with its ability to assess lung aeration and detect complications at the bedside, is increasingly recognized as a valuable tool for prognostic evaluation in various pulmonary conditions. In COVID-19 patients, high LUS aeration scores assessed in the emergency department [50] and in the intermediate care unit [51] were both associated with increased ICU admission and higher mortality rates. One study investigated LUS parameters predictive of clinical deterioration in COVID-19 patients [52]. The presence and percentages of A-lines, discrete B-lines, confluent B-lines, and oxygen saturation were found to have high discriminative accuracy for COVID-19 prognostication. Another large cohort study further explored the prognostic utility of integrating LUS and CT findings for COVID-19 patients [53]. While consolidations on CT and LUS were associated with poor outcomes, and the presence of A-lines in LUS were associated with improved outcomes, the individual imaging findings alone showed limited predictive power. However, when integrated into a nomogram model alongside clinical variables, predictive accuracy significantly improved. These findings highlight the importance of contextualizing LUS findings within the broader clinical picture when assessing prognosis in ARF.

A retrospective multicenter study in ICU invasively ventilated patients investigated both baseline and early changes in LUS aeration scores to predict 30- and 90-day mortality [54]. Baseline LUS scores were not associated with mortality, while early worsening of LUS aeration scores was linked to increased mortality in patients without ARDS. This suggests that dynamic changes in lung aeration assessed by LUS may have greater prognostic value than a single baseline measurement, particularly in critically ill patient populations without ARDS.

## Technical advances and innovation

### Handheld probes

Handheld ultrasound probes represent a major advancement in lung imaging for ARF in the ICU, offering rapid, bedside assessment with improved portability, with greater accessibility, ease of use, and infection control [55]. Comparative studies have shown that handheld ultrasound devices can serve as a reliable alternative to high-end systems in clinical settings, without compromising diagnostic accuracy [56, 57], with concordance rates of 79.3% for B-lines, 88.6% for pleural effusions, and 82.3% for lung consolidations [56]. Additionally, they offer time and cost savings, supporting faster and more efficient patient assessment.

### Machine learning analysis algorithms

Artificial intelligence (AI) integration in critical care LUS has rapidly advanced, especially driven by COVID-19, enabling automated pattern recognition and scoring. Deep learning (DL), particularly convolutional neural networks (CNNs), has been successful in detecting and quantifying important LUS features such as B-lines, pleural effusions, and consolidations.

#### Use of AI in B-line localization

As B-lines provide critical insights into underlying pulmonary pathologies, there is significant interest in developing automated methods for their reliable detection and quantification. One study developed a deep learning algorithm based on a CNN that was trained on phantom and in vivo LUS clips [58]. This LUS analysis algorithm demonstrated high accuracy, sensitivity, and specificity for classifying and localizing B-lines in the in vivo data. Another study used a similar approach to identify B-lines and demonstrated a high correlation between the algorithm’s B-line counts and manual counts from five experts, reporting an intraclass correlation coefficient of 0.84 (95% confidence interval: 0.75–0.90), indicating strong agreement between the automated scoring system and expert assessments [59]. Subsequent research not only localized B-lines, but also effectively quantified their severity with a CNN, offering a more comprehensive assessment of pulmonary involvement [60]. B-line detection remains challenging due to their indistinct, blurred boundaries. A DL model improved automated B-line segmentation by emphasizing precise boundary delineation [61], achieving high specificity (0.97) and precision (0.85) compared to state-of-the-art networks.

#### Automated LUS scoring

AI algorithms have been developed to compute LUS scores by identifying pleural lines, A-lines, B-lines, and consolidations, showing good agreement with expert ratings [62]. The algorithm achieved an 88% agreement with experts for B-line detection, although accuracy dropped when distinguishing isolated from confluent B-lines, especially when estimating whether over 50% of the pleural line was affected, relevant for assigning scores of 1 or 2. Consolidations were identified with a 93% correspondence percentage. This demonstrates that LUS enhanced with AI has a strong potential for clinical use, provided that videos are correctly acquired by experienced sonographers [62]. Another study demonstrated stable agreement between expert and AI LUS scores, with overall moderate agreement between experts and the algorithm [63]. Use of the algorithm demonstrated more consistent scoring, though grading borderline cases remained challenging.

#### Use of AI for pleural effusion detection

Deep learning models including an automated classification algorithm [64], and a CNN combined with a spatial transformer for segmentation [65], have achieved accuracies comparable to experts in detecting and delineating pleural effusions on LUS images. These tools can aid in accurate effusion localization, volume estimation, and safe needle guidance for thoracentesis. However, its effectiveness is limited in poor-quality imaging due to reliance on clear anatomical landmarks.

#### AI-guided LUS by non-experts

A key limitation of LUS is the requirement for operator expertise, requiring dedicated training to adequately acquire and interpret images. Recent studies have explored AI-assisted tools to support non-experts in critical care, improving interpretation accuracy from 68.1% to 93.4% by identifying key LUS patterns such as A-lines, B-lines, consolidations, and pleural effusions [66]. A limitation of this study was that this tool has so far been tested mainly in patients with severe dengue or sepsis. Another study showed that untrained individuals, including COVID-19 patients, could perform LUS with AI support for self-monitoring, highlighting AI’s potential to increase accessibility and reduce operator dependency [67]. Remote AI-guided LUS also reduces healthcare worker exposure in infectious diseases and lowers costs by optimizing staff availability. A recent multicenter validation study found no significant difference in diagnostic quality between AI-supported healthcare professionals and expert sonographers [68], suggesting that AI-driven tools could help broaden access to high-quality LUS in regions with limited availability of experienced practitioners.

## Discussion

LUS has strong clinical utility, but has inherent limitations. It visualizes only the subpleural lung regions through artefact interpretation and does not directly assess deeper parenchyma. Image acquisition and interpretation can be challenging in patients with subcutaneous emphysema, wounds, chest drains, or obesity, where acoustic windows are limited and artefacts may be distorted. [69, 70].

Despite the growing use of LUS in clinical practice, standardized training remains inconsistent, with required instruction time ranging from two to eight hours [71, 72]. AI-based tools that provide real-time guidance and interpretation support [66, 67, 73] may offer a valuable solution to ensure more consistent and accessible training, particularly in resource-limited settings, eliminating inter-observer variability among intensivists. External validation remains necessary to confirm generalizability before widespread clinical adoption. Future research should explore the clinical impact of AI tools in ARF management and address ethical and cultural issues on data privacy, informed consent, and potential algorithmic biases. Additionally, cultural perceptions of AI in clinical decisions may affect acceptance and trust among healthcare providers and patients. A limitation of AI methods is the need for extensive expert annotations, which is time-consuming. To address this, Durrani et al. proposed a method using only 10% of video frames for annotation, enabling accurate classification of lung consolidations in COVID-19 cases [74], reducing labeling effort without compromising performance.

LUS has enabled detailed phenotyping of ARDS, distinguishing focal from non-focal patterns. It remains to be established whether this approach translates into improved patient outcomes, as is currently being investigated in a randomized controlled trial [25, 44]. Standardization of LUS protocols is essential for improving comparability across studies, as most studies currently show high heterogeneity [31]. The recent international expert consensus on quantitative LUS in intensive care represents a major step toward harmonizing definitions and methodologies [7], which may ultimately enhance the consistency and clinical utility of LUS-guided management strategies. Beyond standardization, greater international collaboration is needed to harmonize data collection and facilitate the pooling of multicenter LUS datasets. This would promote innovation, accelerate the development of clinically applicable solutions, and allow for the accurate validation of novel AI tools and segmentation models.

## Conclusions

Bedside LUS has become an essential, radiation-free, portable, and cost-effective tool alternative to traditional imaging for diagnosis and management of ARF. It plays a key role in in ARDS diagnosis and phenotyping, guiding recruitment and fluid strategies, and supporting weaning from mechanical ventilation. Innovations in AI, such as real-time interpretation tools, reduced-label learning, and DL analysis may help overcome operator dependency and training variability. However, standardizing protocols and fostering international collaboration remain essential to validate these tools across diverse populations. Ongoing trials will shed light on whether LUS-guided fluid management and ventilation can improve patient outcomes.

## Data Availability

Data sharing is not applicable to this article as no new datasets were generated or analyzed.
